# Ammonium *O*,*O*′-diethyl dithio­phosphate

**DOI:** 10.1107/S1600536811022811

**Published:** 2011-06-18

**Authors:** Andrzej Okuniewski, Barbara Becker

**Affiliations:** a Department of Inorganic Chemistry, Gdansk University of Technology, 11/12 Narutowicza Str., 80-233 Gdańsk, Poland

## Abstract

In the title compound, NH_4_^+^·(C_2_H_5_O)_2_PS_2_^−^, the ammonium cation is connected by four charge-assisted N—H⋯S hydrogen bonds to four tetra­hedral *O*,*O*′-diethyl dithio­phosphate anions, forming layers parallel to (100). The polar and non-polar constituents of the layers are stacked alternately along [100]. Inter­lacing of the external ethyl groups through van der Waals inter­actions combines these layers into a three-dimensional structure.

## Related literature

For related structures, see: Chekhlov *et al.* (1991 ▶); Chekhlov (2000 ▶). For applications of *O*,*O*′-diethyl dithio­phosphate in coordination chemistry, see: Cotero-Villegas *et al.* (2011 ▶). For the determination of various ions in analytical chemistry using *O*,*O*′-diethyl dithio­phosphates, see: Carletto *et al.* (2009 ▶); Maltez *et al.* (2008 ▶); Pozebon *et al.* (1998 ▶); Wu *et al.* (2006 ▶). For a description of the Cambridge Structural Database, see: Allen (2002 ▶).
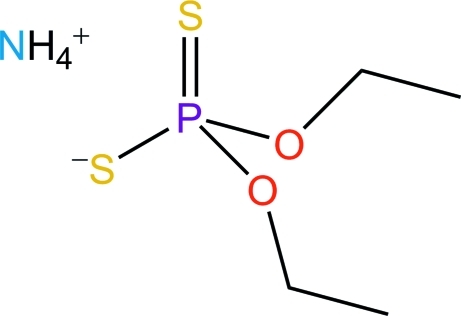


## Experimental

### 

#### Crystal data

NH_4_^+^·C_4_H_10_O_2_PS_2_^−^
*M* *_r_* = 203.25
Monoclinic, 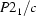

*a* = 12.0274 (7) Å
*b* = 7.2006 (3) Å
*c* = 12.5690 (7) Å
β = 110.305 (6)°
*V* = 1020.89 (9) Å^3^
*Z* = 4
Mo *K*α radiation
μ = 0.63 mm^−1^
*T* = 120 K
0.30 × 0.16 × 0.05 mm

#### Data collection

Oxford Diffraction Xcalibur diffractometer
Absorption correction: analytical [*CrysAlis PRO* (Oxford Diffraction, 2010 ▶) using a multi-faceted crystal model based on expressions derived by Clark & Reid (1995 ▶)] *T*_min_ = 0.856, *T*_max_ = 0.969
3955 measured reflections
2004 independent reflections
1579 reflections with *I* > 2σ(*I*)
*R*_int_ = 0.023

#### Refinement

*R*[*F* ^2^ > 2σ(*F* ^2^)] = 0.035
*wR*(*F* ^2^) = 0.085
*S* = 1.01
2004 reflections
109 parameters
4 restraints
H atoms treated by a mixture of independent and constrained refinement
Δρ_max_ = 0.46 e Å^−3^
Δρ_min_ = −0.21 e Å^−3^

### 

Data collection: *CrysAlis PRO* (Oxford Diffraction, 2010 ▶); cell refinement: *CrysAlis PRO*; data reduction: *CrysAlis PRO*; program(s) used to solve structure: *SHELXS97* (Sheldrick, 2008 ▶); program(s) used to refine structure: *SHELXL97* (Sheldrick, 2008 ▶); molecular graphics: *OLEX2* (Dolomanov *et al.*, 2009 ▶) and *Mercury* (Macrae *et al.*, 2008 ▶); software used to prepare material for publication: *WinGX* (Farrugia, 1999 ▶) and *PLATON* (Spek, 2009 ▶).

## Supplementary Material

Crystal structure: contains datablock(s) global, I. DOI: 10.1107/S1600536811022811/wm2499sup1.cif

Structure factors: contains datablock(s) I. DOI: 10.1107/S1600536811022811/wm2499Isup2.hkl

Supplementary material file. DOI: 10.1107/S1600536811022811/wm2499Isup3.cml

Additional supplementary materials:  crystallographic information; 3D view; checkCIF report

## Figures and Tables

**Table 1:** Hydrogen-bond geometry (Å, °)

*D*—H⋯*A*	*D*—H	H⋯*A*	*D*⋯*A*	*D*—H⋯*A*
N1—H1*N*⋯S1^i^	0.89 (1)	2.43 (1)	3.310 (2)	178 (3)
N1—H4*N*⋯S1^ii^	0.88 (1)	2.50 (1)	3.377 (2)	177 (2)
N1—H3*N*⋯S2^iii^	0.89 (1)	2.54 (1)	3.409 (2)	169 (2)
N1—H2*N*⋯S2	0.88 (1)	2.39 (1)	3.2633 (19)	171 (2)

Symmetry codes: (i) 
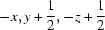
; (ii) 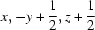; (iii) 
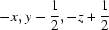
.

**Fig. 1.**
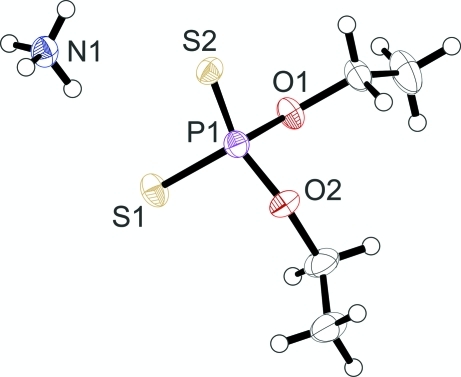
The molecular structure of NH4+.(C2H5O)2PS2-, with displacement ellipsoids drawn at the 50% probability level.

**Fig. 2.**
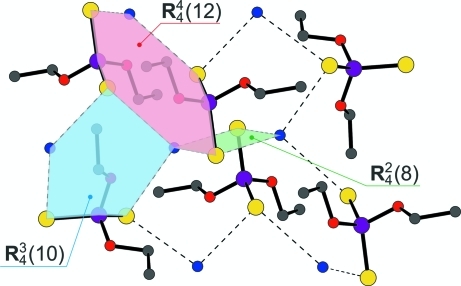
Hydrogen bond pattern found in the structure of NH4+.(C2H5O)2PS2-, with R24(8), R44(12) and R34(10) structural motifs depicted. Hydrogen bonds are marked with dashed lines.

**Fig. 3.**
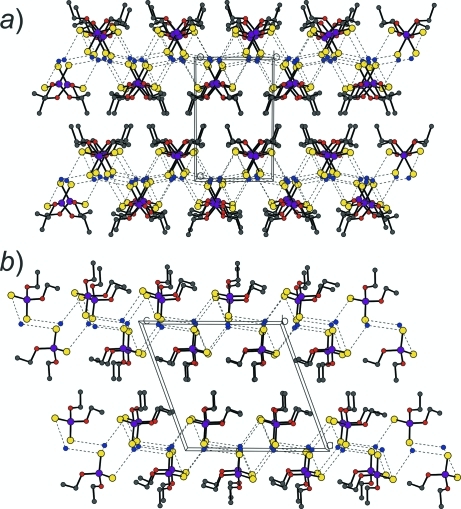
Layers of NH4+.(C2H5O)2PS2- projected down [001] (a)) and [010] (b)). Hydrogen bonds are marked with dashed lines, with hydrogen atoms omitted for clarity.

## supplementary crystallographic information

### Comment

Ammonium *O*,*O*'-diethyl dithiophosphate is frequently used as a source of the (C_2_H_5_O)_2_PS_2_^-^ ligand in coordination chemistry (Cotero-Villegas *et al.*, 2011) and in analytical chemistry for determination of various ions, *eg.* As (Pozebon *et al.*, 1998), Pb (Maltez *et al.*, 2008), Cd (Carletto *et al.*, 2009), Hg (Wu *et al.*, 2006).

There are at least 340 structures deposited in the Cambridge Structural Database (v5.32; Allen, 2008) containing the *O*,*O*'-diethyl dithiophosphate moiety, but there are no crystal structure of simple ammonium, sodium or potassium salts reported. Among these structures one can find 328 complexes (including 204 of row 6 family metals, mainly molybdenum compounds), five compounds with complex cations, five simple organic or inorganic compounds and finally two salts of 1,10-diaza-18-crown-6 (Chekhlov, 2000; Chekhlov *et al.*, 1991).

In the crystal structure of the title compound, the asymmetric unit consists of one ammonium cation and one tetrahedral *O*,*O*'-diethyl dithiophosphate anion (Fig. 1). The P—S distances are 1.9720 (8) Å and 1.9753 (8) Å. These values are slightly lower than the mean value of 1.9872 (25) Å calculated for the 340 compounds deposited in the CSD. Each ammonium cation is connected by four N—H···S hydrogen bonds to four *O*,*O*'-diethyl dithiophosphate anions. This way three structural ring motifs are formed: two of them are centrosymmetric – R^2^_4_(8), R^4^_4_(12), and one is not – R^3^_4_(10) (Fig. 2.) Hydrogen bonding interactions are summarized in Tab. 1.

The connected ions form layers parallel to the (100) plane. Each layer has an hydrophilic interior, where heteroatoms and hydrogen bonds can be found, and an hydrophobic exterior formed by the ethyl groups (Fig. 3). These layers interact with each other by van der Waals forces forming a three-dimensional crystal structure.

### Experimental

1 g of commercially available ammonium *O*,*O*'-diethyl dithiophosphate was dissolved in 5 ml of acetone and left to evaporate slowly. After one week colourless crystals suitable for single-crystal X-ray diffraction analysis were collected.

### Refinement

Hydrogen atoms were placed at the calculated positions (*d*_CH_ = 0.98–0.99 Å) and were treated as riding on their parent atoms, with *U*(H) set to 1.2–1.5 times *U*_eq_(C). The N—H distances were restrained to 0.88 (1) Å.

### Crystal data

**Table:**

NH_4_^+^·C_4_H_10_O_2_PS_2_^−^	*F*(000) = 432
*M**_r_* = 203.25	*D*_x_ = 1.322 Mg m^−^^3^
Monoclinic, *P*2_1_/*c*	Melting point: 438(1) K
Hall symbol: -P 2ybc	Mo *K*α radiation, λ = 0.71073 Å
*a* = 12.0274 (7) Å	Cell parameters from 2402 reflections
*b* = 7.2006 (3) Å	θ = 2.8–28.4°
*c* = 12.5690 (7) Å	µ = 0.63 mm^−^^1^
β = 110.305 (6)°	*T* = 120 K
*V* = 1020.89 (9) Å^3^	Plate, colourless
*Z* = 4	0.30 × 0.16 × 0.05 mm

### Data collection

**Table:**

Oxford Diffraction Xcalibur diffractometer	2004 independent reflections
graphite	1579 reflections with *I* > 2σ(*I*)
Detector resolution: 8.1883 pixels mm^-1^	*R*_int_ = 0.023
ω scans	θ_max_ = 26.0°, θ_min_ = 3.3°
Absorption correction: analytical [*CrysAlis PRO* (Oxford Diffraction, 2010) using a multi-faceted crystal model based on expressions derived by Clark & Reid (1995)]	*h* = −14→12
*T*_min_ = 0.856, *T*_max_ = 0.969	*k* = −8→8
3955 measured reflections	*l* = −15→14

### Refinement

**Table:**

Refinement on *F*^2^	Primary atom site location: structure-invariant direct methods
Least-squares matrix: full	Secondary atom site location: difference Fourier map
*R*[*F*^2^ > 2σ(*F*^2^)] = 0.035	Hydrogen site location: inferred from neighbouring sites
*wR*(*F*^2^) = 0.085	H atoms treated by a mixture of independent and constrained refinement
*S* = 1.01	*w* = 1/[σ^2^(*F*_o_^2^) + (0.0521*P*)^2^] where *P* = (*F*_o_^2^ + 2*F*_c_^2^)/3
2004 reflections	(Δ/σ)_max_ = 0.001
109 parameters	Δρ_max_ = 0.46 e Å^−^^3^
4 restraints	Δρ_min_ = −0.21 e Å^−^^3^

### Special details

**Table:**

Geometry. All s.u.'s (except the s.u. in the dihedral angle between two l.s. planes) are estimated using the full covariance matrix. The cell s.u.'s are taken into account individually in the estimation of s.u.'s in distances, angles and torsion angles; correlations between s.u.'s in cell parameters are only used when they are defined by crystal symmetry. An approximate (isotropic) treatment of cell s.u.'s is used for estimating s.u.'s involving l.s. planes.
Refinement. Refinement of *F*^2^ against ALL reflections. The weighted *R*-factor *wR* and goodness of fit *S* are based on *F*^2^, conventional *R*-factors *R* are based on *F*, with *F* set to zero for negative *F*^2^. The threshold expression of *F*^2^ > 2σ(*F*^2^) is used only for calculating *R*-factors(gt) *etc*. and is not relevant to the choice of reflections for refinement. *R*-factors based on *F*^2^ are statistically about twice as large as those based on *F*, and *R*- factors based on ALL data will be even larger.

### Fractional atomic coordinates and isotropic or equivalent isotropic displacement parameters (Å^2^)

**Table:**

	*x*	*y*	*z*	*U*_iso_*/*U*_eq_	
C1	0.3199 (2)	0.5211 (3)	0.2964 (2)	0.0326 (5)	
H1A	0.2982	0.4906	0.3635	0.039*	
H1B	0.265	0.6179	0.2517	0.039*	
C2	0.4451 (2)	0.5890 (4)	0.3330 (3)	0.0511 (7)	
H2A	0.4984	0.493	0.3783	0.077*	
H2B	0.4529	0.7018	0.3788	0.077*	
H2C	0.466	0.6169	0.266	0.077*	
C3	0.3169 (2)	0.0606 (4)	0.3984 (2)	0.0392 (6)	
H3A	0.369	0.1581	0.4457	0.047*	
H3B	0.36	−0.0022	0.3543	0.047*	
C4	0.2849 (3)	−0.0763 (4)	0.4717 (2)	0.0425 (7)	
H4A	0.2426	−0.0129	0.5152	0.064*	
H4B	0.3572	−0.1334	0.524	0.064*	
H4C	0.234	−0.1727	0.4242	0.064*	
O1	0.31244 (13)	0.35602 (19)	0.22740 (13)	0.0281 (4)	
O2	0.20831 (13)	0.1435 (2)	0.32198 (12)	0.0278 (4)	
P1	0.20257 (5)	0.21895 (8)	0.20040 (5)	0.02272 (16)	
S1	0.23286 (5)	0.02043 (8)	0.10585 (5)	0.03012 (17)	
S2	0.04716 (5)	0.34439 (8)	0.13990 (4)	0.02542 (16)	
N1	0.0076 (2)	0.3099 (3)	0.38320 (17)	0.0284 (4)	
H1N	−0.0563 (17)	0.369 (4)	0.385 (2)	0.053 (8)*	
H2N	0.020 (2)	0.333 (4)	0.3191 (14)	0.047 (8)*	
H3N	−0.010 (3)	0.1909 (16)	0.385 (2)	0.052 (9)*	
H4N	0.0644 (18)	0.357 (3)	0.4414 (15)	0.047 (8)*	

### Atomic displacement parameters (Å^2^)

**Table:**

	*U*^11^	*U*^22^	*U*^33^	*U*^12^	*U*^13^	*U*^23^
C1	0.0318 (13)	0.0262 (11)	0.0376 (13)	−0.0028 (10)	0.0093 (10)	−0.0092 (10)
C2	0.0360 (15)	0.0437 (15)	0.0672 (19)	−0.0085 (13)	0.0101 (14)	−0.0231 (15)
C3	0.0281 (13)	0.0406 (14)	0.0387 (14)	0.0043 (12)	−0.0015 (11)	0.0107 (12)
C4	0.0528 (17)	0.0459 (15)	0.0302 (13)	0.0196 (14)	0.0162 (12)	0.0099 (12)
O1	0.0263 (8)	0.0254 (8)	0.0348 (8)	−0.0061 (7)	0.0133 (7)	−0.0074 (7)
O2	0.0250 (8)	0.0333 (8)	0.0231 (7)	0.0047 (7)	0.0061 (6)	0.0067 (7)
P1	0.0227 (3)	0.0229 (3)	0.0233 (3)	−0.0008 (2)	0.0088 (2)	−0.0015 (2)
S1	0.0256 (3)	0.0295 (3)	0.0370 (3)	−0.0010 (2)	0.0131 (3)	−0.0097 (3)
S2	0.0263 (3)	0.0297 (3)	0.0202 (3)	0.0051 (2)	0.0080 (2)	0.0011 (2)
N1	0.0328 (12)	0.0313 (12)	0.0244 (10)	0.0047 (10)	0.0141 (9)	0.0026 (9)

### Geometric parameters (Å, °)

**Table:**

C1—O1	1.456 (3)	C4—H4A	0.98
C1—C2	1.495 (3)	C4—H4B	0.98
C1—H1A	0.99	C4—H4C	0.98
C1—H1B	0.99	O1—P1	1.5888 (15)
C2—H2A	0.98	O2—P1	1.6005 (14)
C2—H2B	0.98	P1—S1	1.9720 (8)
C2—H2C	0.98	P1—S2	1.9753 (8)
C3—O2	1.454 (3)	N1—H1N	0.886 (10)
C3—C4	1.489 (3)	N1—H2N	0.884 (10)
C3—H3A	0.99	N1—H3N	0.885 (10)
C3—H3B	0.99	N1—H4N	0.879 (10)
			
O1—C1—C2	107.3 (2)	C3—C4—H4B	109.5
O1—C1—H1A	110.2	H4A—C4—H4B	109.5
C2—C1—H1A	110.2	C3—C4—H4C	109.5
O1—C1—H1B	110.2	H4A—C4—H4C	109.5
C2—C1—H1B	110.2	H4B—C4—H4C	109.5
H1A—C1—H1B	108.5	C1—O1—P1	120.63 (14)
C1—C2—H2A	109.5	C3—O2—P1	120.07 (15)
C1—C2—H2B	109.5	O1—P1—O2	104.47 (8)
H2A—C2—H2B	109.5	O1—P1—S1	105.37 (6)
C1—C2—H2C	109.5	O2—P1—S1	111.94 (6)
H2A—C2—H2C	109.5	O1—P1—S2	113.83 (6)
H2B—C2—H2C	109.5	O2—P1—S2	104.14 (6)
O2—C3—C4	108.3 (2)	S1—P1—S2	116.59 (4)
O2—C3—H3A	110	H1N—N1—H2N	111 (2)
C4—C3—H3A	110	H1N—N1—H3N	104 (3)
O2—C3—H3B	110	H2N—N1—H3N	109 (2)
C4—C3—H3B	110	H1N—N1—H4N	103 (2)
H3A—C3—H3B	108.4	H2N—N1—H4N	111 (3)
C3—C4—H4A	109.5	H3N—N1—H4N	118 (3)

### Hydrogen-bond geometry (Å, °)

**Table:**

*D*—H···*A*	*D*—H	H···*A*	*D*···*A*	*D*—H···*A*
N1—H1N···S1^i^	0.89 (1)	2.43 (1)	3.310 (2)	178 (3)
N1—H4N···S1^ii^	0.88 (1)	2.50 (1)	3.377 (2)	177 (2)
N1—H3N···S2^iii^	0.89 (1)	2.54 (1)	3.409 (2)	169 (2)
N1—H2N···S2	0.88 (1)	2.39 (1)	3.2633 (19)	171 (2)

Symmetry codes: (i) −*x*, *y*+1/2, −*z*+1/2; (ii) *x*, −*y*+1/2, *z*+1/2; (iii) −*x*, *y*−1/2, −*z*+1/2.
